# Long-pulsed alexandrite laser depilation of hard palate radial forearm free flap reconstruction

**DOI:** 10.1016/j.jdcr.2023.11.011

**Published:** 2023-11-25

**Authors:** Nishita Lockwood, Donovan Lockwood, Ansley Roche, Saral Mehra, Kathleen Suozzi

**Affiliations:** a Department of Dermatology, Yale New Haven Hospital, New Haven, Connecticut; b Division of Otolaryngology-Head and Neck Surgery, Department of Surgery, Yale New Haven Hospital, New Haven, Connecticut

**Keywords:** hair removal, intraoral reconstruction, lasers

## Introduction

Although there have been limited reports of the use of laser hair removal techniques in free flap reconstruction, to our knowledge there is a relative dearth of information on the use of intraoral laser hair removal. We report an excellent result using long-pulsed alexandrite (LP-alexandrite) laser depilation on an *in situ* hard palate radial forearm free flap reconstruction.

Microvascular free tissue transfer, or free flaps, are a commonly used and valuable tool in reconstruction after resection of head and neck malignancies. One of the most commonly used donor sites is the radial forearm, which can bear thick terminal hairs especially in male patients.^1^ In cases where radiation is necessary, destruction of hair follicles commonly occurs and no further treatment is necessary.^2^ In patients who do not require radiation, hair can continue to grow on the reconstruction site postoperatively and cause undesirable sequelae such as dysphagia, salivary pooling, discomfort, and poor aesthetic outcome.^3^^,^^4^ Hair-bearing flap reconstructions can also complicate oncologic surveillance, making management of hair an important consideration following reconstruction.

## Case report

A 37-year-old male former smoker presented to Head and Neck Surgical Oncology with a biopsy proven mucoepidermoid carcinoma of the hard palate. He underwent partial palatectomy, including soft tissue and bone resection with negative margins, which resulted in an oroantral fistula. After a discussion of reconstructive options including soft tissue reconstruction versus obturator placement, the patient elected reconstruction with a radial forearm free flap and ipsilateral neck dissection. At his 3- and 6-month follow-up appointments, the patient noted concern of thick, quick-growing dark hair growing on his hard palate, which induced gagging and discomfort. He was then referred to Dermatology for consideration of laser hair removal.

At his initial dermatology examination, he was noted to have a 3-cm hypopigmented patch with approximately 20 long, dark terminal hairs. The patient was skin type II with dark brown hair. He was treating this bothersome hair with tweezing and trimming but desired a permanent solution.

**Fig 1:**
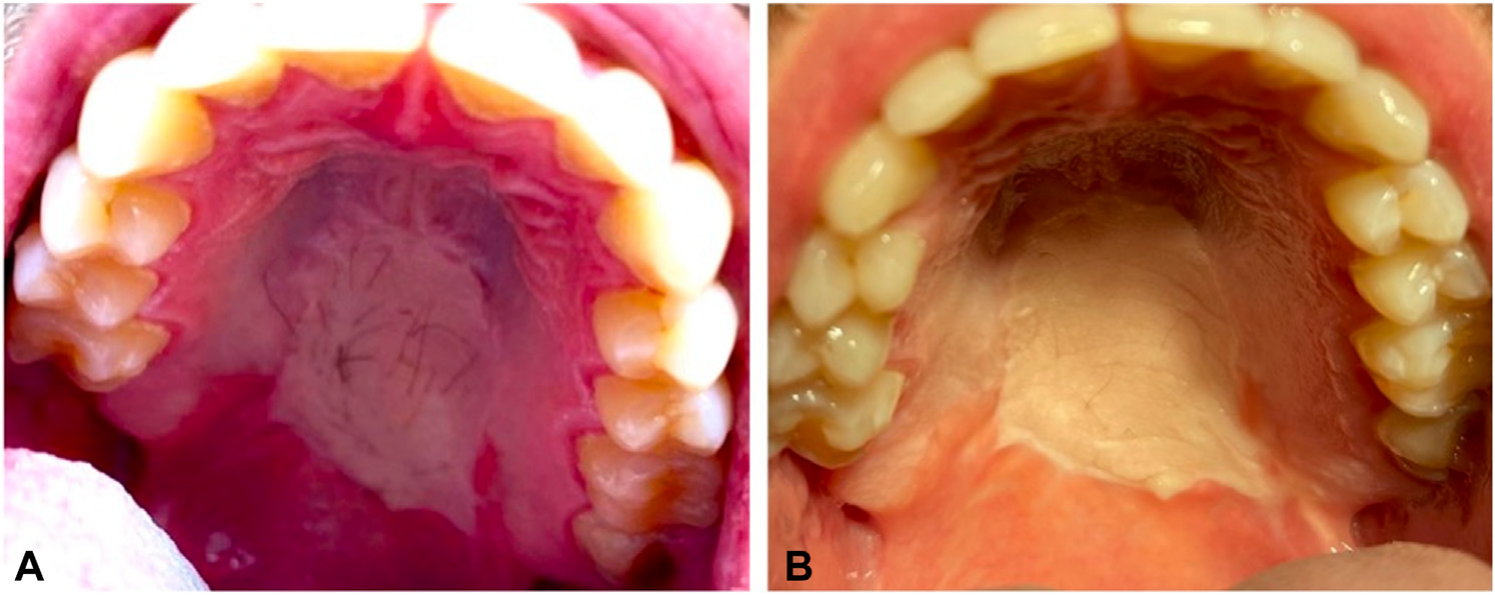
Results of laser hair removal treatment for intraoral terminal hairs secondary to transposed free flap from forearm to hard palate. **A,** Pretreatment; **B,** posttreatment long-pulsed alexandrite × 2 treatments.

**Fig 2:**
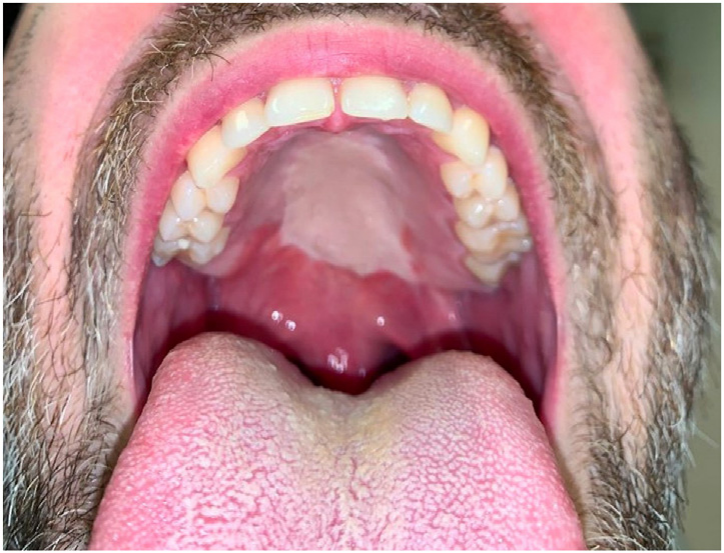
After completion of treatment.

We elected to attempt treatment with intraoral LP-alexandrite laser. Treatment was carried out at 6-week intervals with LP-alexandrite laser (GentleMax, Candela Corporation), 755 nm, 24 J/cm^2^, 30 ms pulse duration, 15 mm spot size. Fig 1 shows the clinical findings before treatment (Fig 1, *A*) and after 2 treatment sessions (Fig 1, *B*). A third session was performed, resulting in complete hair removal (Fig 2).The laser handpiece was protected with a sterile plastic shield before insertion into the patient’s oral cavity. The device was able to reach the full extent of the involved area of the palate. No anesthesia was required, and the patient tolerated the procedure well.

## Discussion

Oral cavity and pharyngeal cancers comprise 3% of the newly diagnosed cancers in the United States per year.^5^ Reconstruction with free flaps is a common technique used after resection of oral and oropharyngeal tumors, however, flaps with hair follicles can lead to several adverse outcomes that impact quality of life. For this reason, it is critical to find effective and safe techniques to reduce hair growth following reconstruction.

Long-pulsed diode, alexandrite, intense pulsed light, and Nd:YAG lasers have all been reported as effective long-term solutions for hair removal.^6^ Some studies have found however that LP-alexandrite lasers result in better reduction in hair count.^7^ In our patient, we found that the LP-alexandrite system was efficacious and did not cause significant discomfort without the use of any anesthesia technique. This specific laser was chosen given our patient was skin type II with dark hair and higher fluences can be used to achieve results in lower number of sessions.

There have been several reports of the use of laser hair removal on free flap reconstructions. In a 2013 case series from Germany, intraoral Nd:YAG laser hair removal was performed with good result in oral cavity and oropharyngeal sites, although the authors performed this under general anesthesia with nasotracheal intubation due to concerns for access with the laser handpiece.^8^ Another group out of Birmingham, United Kingdom reported a case series of 5 patients, 4 of which had successful intraoral hair removal with LP-alexandrite laser but 1 unsuccessful case which was attributed to difficult access in the oral cavity.^1^ Notably, many of the reported cases include oral tongue or floor of mouth reconstruction sites, which may be more easily accessed in the office than the hard palate or oropharyngeal sites. There are certain safety considerations regarding laser in the oral cavity. The mucosal surface is more prone to erosions after laser treatment. Starting settings should be adjusted to lower fluence than would be used on nonmucosal skin. That said, the grafted skin in the mouth has retained its follicles making it inherently closer to nonmucosal skin and in this patient average fluence settings were tolerated well without complication. There was not targeting by the laser of mucosal or dental surfaces outside of the flap region.

It is worth noting that preoperative depilation could be considered in good candidates, given that the planning did not significantly delay oncologic treatment. Additionally, there is often some uncertainty about which free flap reconstruction method (radial forearm, anterolateral thigh, etc) will be used. Further research is necessary to determine optimal time between depilation therapy and free flap harvest/inset.

We report that intraoral laser hair removal is a cost effective, safe, and easy technique which can be performed in the office without the need for anesthesia or an operating room.

Overall, this procedure is highly generalizable for many skin types and hair colors. The only hair color that cannot be targeted is white hair. Potential limitations include adequate anesthesia and location of flap. This patient had no tissue sensation in the reconstruction and had no discomfort during the procedure. The use of injected or topical anesthesia in this area is possible albeit could be technically challenging; however, local anesthesia could be readily used for other body surfaces. In addition, the posterior border of the flap in this patient was difficult to access with the laser hand piece and this could lead to difficulty in some patients. Overall, the patient had a successful result seen in Fig 2.

## Conflicts of interest

None disclosed.

## References

[1] Shim T.N., Abdullah A., Lanigan S., Avery C. (2011). Hairy intraoral flap – an unusual indication for laser epilation: a series of 5 cases and review of the literature. Br J Oral Maxillofac Surg.

[2] Maisel R.H., Liston S.L., Adams G.L. (1983). Complications of pectoralis myocutaneous flaps. Laryngoscope.

[3] Kwak D.L., Kattan K.R. (1983). Hair growing in the esophagus: complication of reconstruction of the pharynx and esophagus. South Med J.

[4] McLean G., Laufer I. (1979). Hairy esophagus: a complication of pharyngoesophageal reconstructive surgery in two cases. AJR Am J Roentgenol.

[5] Ellington T.D., Henley S.J., Senkomago V. (2020). Trends in incidence of cancers of the oral cavity and pharynx — United States 2007–2016. MMWR Morb Mortal Wkly Rep.

[6] Gan S.D., Graber E.M. (2013). Laser hair removal: a review. Dermatol Surg.

[7] Dorgham N.A., Dorgham D.A. (2020). Lasers for reduction of unwanted hair in skin of colour: a systematic review and meta-analysis. J Eur Acad Dermatol Venereol.

[8] Kaune K.M., Haas E., Jantke M. (2013). Successful Nd:YAG laser therapy for hair removal in the oral cavity after plastic reconstruction using hairy donor sites. Dermatology.

